# Machine learning analysis to predict the need for ankle foot orthosis in patients with stroke

**DOI:** 10.1038/s41598-021-87826-3

**Published:** 2021-04-19

**Authors:** Yoo Jin Choo, Jeoung Kun Kim, Jang Hwan Kim, Min Cheol Chang, Donghwi Park

**Affiliations:** 1 Department of Physical Medicine and Rehabilitation, College of Medicine, Yeoungnam University, 317-1, Daemyungdong, Namku, Daegu, 705-717 Republic of Korea; 2 Department of Business Administration, School of Business, Yeungnam University, Gyeongsan-si, Republic of Korea; 3 Department of Biomedical Engineering and Welfare Technology, Hanseo University, Seosan, Chungnam Province Republic of Korea; 4 Department of Physical Medicine and Rehabilitation, Ulsan University Hospital, University of Ulsan College of Medicine, 877 Bangeojinsunghwndo-ro, Dong-gu, Ulsan, 44033 Republic of Korea

**Keywords:** Neurology, Neurological disorders

## Abstract

We investigated the potential of machine learning techniques, at an early stage after stroke, to predict the need for ankle–foot orthosis (AFO) in stroke patients. We retrospectively recruited 474 consecutive stroke patients. The need for AFO during ambulation (output variable) was classified according to the Medical Research Council (MRC) score for the ankle dorsiflexor of the affected limb. Patients with an MRC score of < 3 for the ankle dorsiflexor of the affected side were considered to require AFO, while those with scores ≥ 3 were considered not to require AFO. The following demographic and clinical data collected when patients were transferred to the rehabilitation unit (16.20 ± 6.02 days) and 6 months after stroke onset were used as input data: age, sex, type of stroke (ischemic/hemorrhagic), motor evoked potential data on the tibialis anterior muscle of the affected side, modified Brunnstrom classification, functional ambulation category, MRC score for muscle strength for shoulder abduction, elbow flexion, finger flexion, finger extension, hip flexion, knee extension, and ankle dorsiflexion of the affected side. For the deep neural network model, the area under the curve (AUC) was 0.887. For the random forest and logistic regression models, the AUC was 0.855 and 0.845, respectively. Our findings demonstrate that machine learning algorithms, particularly the deep neural network, are useful for predicting the need for AFO in stroke patients during the recovery phase.

## Introduction

Stroke is a leading cause of serious long-term disability in the adult population, and it is the second leading cause of death of the elderly in high-income countries^1^. Most patients with stroke suffer from lower limb hemiparesis, which disturbs gait function, and of them, more than half are reported to have gait problems in the chronic stage of stroke^2, 3^.

Weakness on ankle dorsiflexion is one of the major causes of gait disturbance after stroke, which results in instability of the ankle during the stance phase and reduced clearance during the swing phase^4, 5^. For patients with weakness on ankle dorsiflexion, an ankle–foot orthosis (AFO) is commonly applied, which can provide medial–lateral stability at the ankle during the stance phase, and improve clearance during the swing phase^4, 6^.

However, in our clinical practice, we often experience the following scenario: for a patient with motor weakness in the ankle dorsiflexor (e.g. Medical Research Council [MRC]: grade 1–2) 1 month after stroke, the clinician prescribes an AFO. However, 2 weeks later, the strength of the patient’s ankle dorsiflexor may improve to MRC grade 4. Consequently, continued use of the AFO is not necessary. This patient may consider this situation as a waste of money.

Stroke recovery is relatively rapid during the first month after stroke onset, but continues at a slower pace between 3 and 6 months^7^. Moreover, only minor improvements in the recovery of motor function occur 6 months after stroke onset^8^. Therefore, for determining the continuous necessity of orthoses, clinicians should predict the motor function of patients at ≥ 6 months after stroke onset.

Accurate and early prediction of the recovery of ankle dorsiflexion strength may help to reduce the prevalence of unnecessary AFO use in stroke patients. However, to date, there has been no tool for predicting the necessity of the use of AFO. With recent developments in technology, new techniques such as machine learning have been used to assist clinicians in predicting patients’ motor recovery^9^. Machine learning is a technique in artificial intelligence (AI) in which a system learns patterns and rules from given information. Machine learning has several advantages regarding the detection of possible interactions between many attributes/variables and hence may be useful in clinical prediction^9–11^. In previous studies, machine learning techniques have been used to predict motor and functional recovery in the acute and subacute stages of stroke^12–16^. However, to date, no machine learning study has investigated the prediction of the need for AFO in stroke patients. Therefore, considering its expected impact on stroke management, this study aimed to apply machine learning to predict the need for AFO in stroke patients.

## Methods

**Figure 1:**
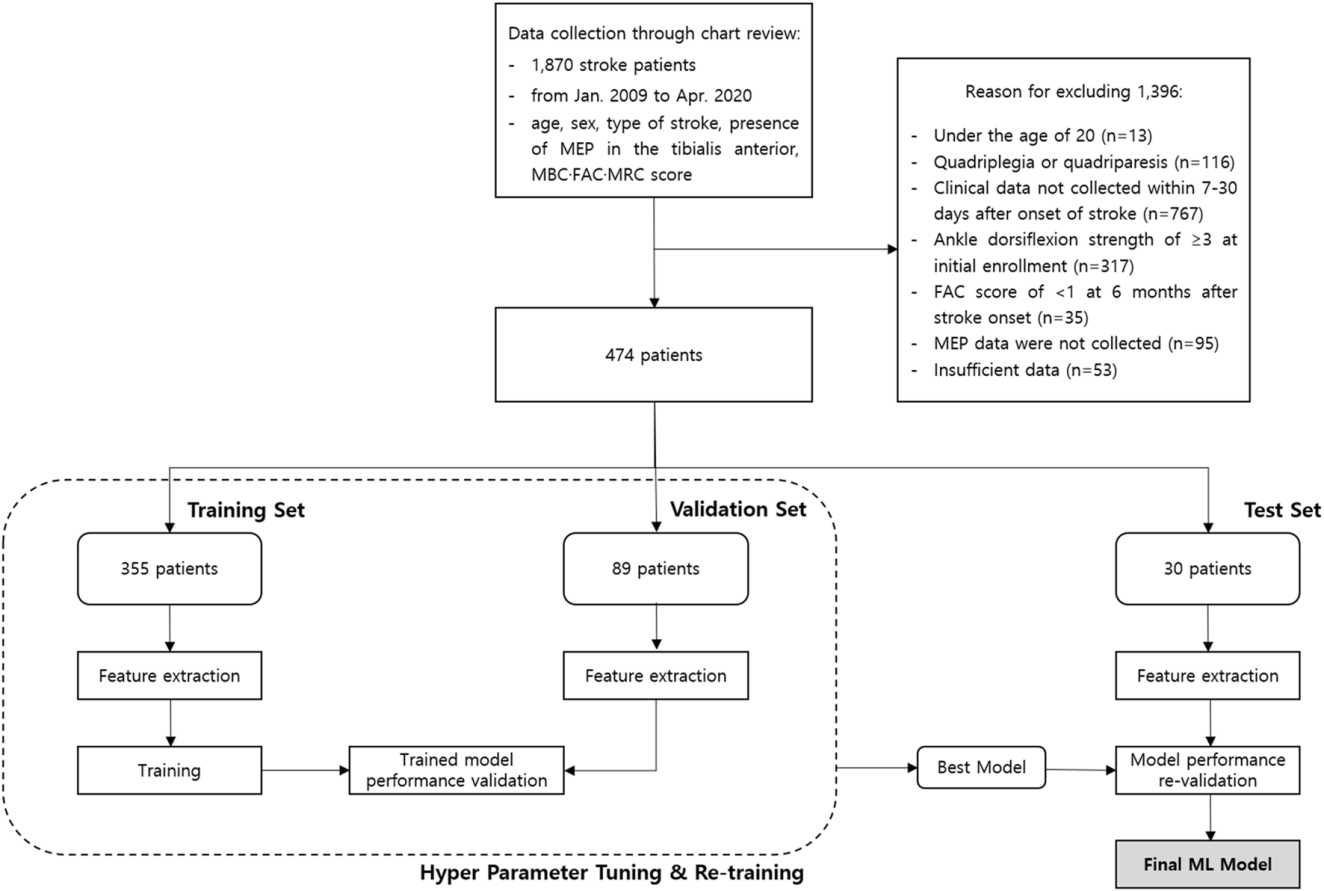
The overall modeling process of this study. *MEP* motor evoked potential, *MBC* modified Brunnstrom classification, *FAC* functional ambulation category, *MRC* medical research council, *ML* machine learning.

This study was approved by the Institutional Review Board of Yeungnam University hospital, and informed consent was waived because of the retrospective nature of the study and because the analysis involved anonymous clinical data. All methods were carried out in accordance with relevant guidelines and regulations. This study included patients who were admitted to the rehabilitation department of a single university hospital because of stroke and who were diagnosed using magnetic resonance imaging from January 2009 to April 2020. The steps of the modeling process applied in this study are shown in Fig. 1.

### Data collection

The inclusion criteria were as follows: (1) first-ever stroke; (2) age over 20 years; (3) hemiplegia or hemiparesis following stroke; (4) clinical data collected within 7–30 days (early stage, day of transfer, or day of admission to the rehabilitation department) after onset; (5) absence of serious medical complications, such as pneumonia or cardiac problems from onset to final evaluation; and (6) presence of a functional ambulation category (FAC) score of ≥ 1 at 6 months after stroke onset. The exclusion criteria were as follows: (1) ankle dorsiflexion strength of ≥ 3 at initial enrollment; (2) other preexisting brain or spinal cord lesions; and (3) presence of other peripheral neuropathies that could affect ankle dorsiflexion strength, such as peripheral polyneuropathy.

The following demographic and clinical data were collected when patients were transferred to the rehabilitation unit (16.2 ± 6.0 days after stroke onset): age, sex, type of stroke (ischemic/hemorrhagic), the presence of motor evoked potential (MEP) data for the tibialis anterior muscle of the affected side, modified Brunnstrom classification (MBC), FAC, and MRC score for muscle strength with respect to shoulder abduction, elbow flexion, finger flexion, finger extension, hip flexion, knee extension, and ankle dorsiflexion of the affected side. We have selected these input variables because they represent clinical data that is commonly collected when stroke patients are admitted or visit the hospital for rehabilitation. Regarding MEP evaluation, transcranial magnetic stimulation was performed using a Magstim Novametrix 200 magnetic stimulator (Novametrix Inc., Wallingford, CT, USA) with a circular coil (7-cm mean diameter). While the patients were in a relaxed state, MEPs were recorded from tibialis anterior. Details of the other stimulation methods have been outlined in a previous study^17^. Moreover, we determined the MRC score of ankle dorsiflexion for the affected side at 6 months after stroke onset.

We used 3 machine learning algorithms: deep neural network, random forest, and logistic regression^14^. The deep neural network consists of layers of interconnected artificial neurons. An artificial neuron is designed based on the biological neuron and receives multiple inputs multiplied by weights, and outputs the sum of the inputs^18^. The random forest algorithm comprises several decision trees that consist of multiple true or false conditions using input variables^19^. The sum of the decisions made by the decision trees is used for the final classification^19^. The machine learning models were trained with all variables as inputs to classify patients that were likely to require AFO for the lower extremity of the affected side. For the deep neural network model, 4 layers with 256–512-1024–512 neurons, RMSProp optimizer, and relu activation were used. For the random forest model, 500 decision trees were used. We categorized the output variables as the necessity and non-necessity of AFO during ambulation. Patients with an MRC score of < 3 for the ankle dorsiflexor of the affected side were considered to require AFO, while patients with scores of ≥ 3 were considered not to require AFO.

To prevent overfitting, we reduced the network size (only 4 layers), applied dropout regulation and early stopping, and held back validation and test datasets to check potential overfitting. To avoid under-fitting, we used neural networks with the capability of capturing the variability of the training dataset.

Of the study population, 75% (n = 335), 18.75% (n = 89), and 6.25% (n = 30) were included in the training, validation, and test sets, respectively, to prevent overfitting of the models. TensorFlow version 1.1.0 (Google, Mountain View, CA) and scikit-learn toolkit version 0.18.1 (Google) were used to train the machine learning models.

### Statistical analysis

Statistical analyses were performed using python 3.7.9 and scikit-learn version 0.23.2. Receiver operating characteristic curve analysis was employed, and the area under the curve (AUC) was calculated. The confidence interval (CI) for the AUC was calculated using the approach used by DeLong et al^20^.

## Results

**Figure 2:**
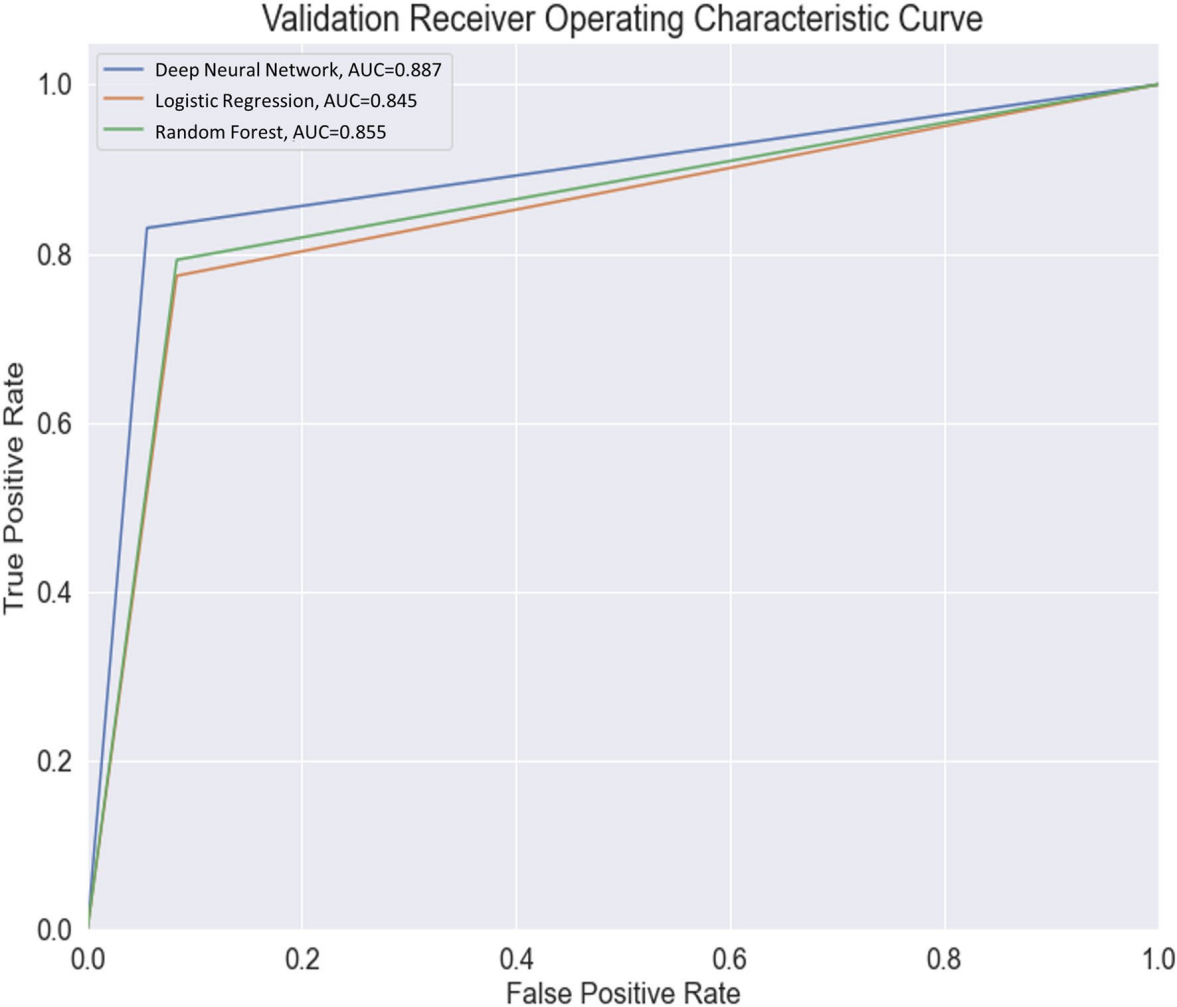
Receiver operating characteristic curve for the models for data validation. The deep neural network model is superior with an area under the curve of 0.887, followed by the random forest model with an area under the curve of 0.855 and the logistic regression model with an area under the curve of 0.845. AUC: area under the curve.

A total of 474 patients (mean age 60.3 ± 12.8 years; 269 males, 205 females) were included in this study (Table 1). Of the 474 patients, 193 (40.7%) required AFO (ankle dorsiflexor MRC score < 3), while 281 (59.3%) did not need AFO (Table 1). The AUC of the validation dataset for the deep neural network model was 0.887 [95% CI, 0.824–0.951]. For the random forest and logistic regression models, the AUC was 0.855 [95% CI, 0.783–0.926] and 0.845 [95% CI, 0.772–0.918], respectively (Table 2) (Fig. 2).

**Table 1:** The demographic data of the stroke patients included in this study.

Variables	Results
**Demographic data**	
Number of patients, n	474
Age, years	60.3 ± 12.8
Days to transfer or admission	16.2 ± 6.0
**Clinical data (when patients were transferred to the rehabilitation unit, 16.2 ± 6.0 days after stroke onset)**	
MBC	1.8 ± 1.4
FAC	0.4 ± 0.7
MRC	
Shoulder abductor	1.0 ± 1.2
Elbow flexor	1.0 ± 1.3
Finger flexor	0.8 ± 1.2
Finger extensor	0.7 ± 1.2
Hip flexor	1.2 ± 1.1
Knee extensor	1.2 ± 1.2
Ankle dorsiflexor	0.5 ± 0.8
**The presence of MEP (presence : absence, n)**	
Tibialis anterior	185 : 289

*MBC* modified Brunnstrom classification; *FAC* functional ambulation category; *MRC* medical research council; *MEP* motor evoked potential.

**Table 2:** Outcomes of the three prediction models.

ML model	Prediction model
Sample size (patients)	355 for training, 89 for validation, 30 for test, total 474
Sample zero ratio	Train 40.8%, validation 40.5%, test 40.0%
DNN	- 4 layers with 256–512-1024–512 neurons, RMSProp optimizer, relu activation - Training accuracy: 79.7% - Validation accuracy: 87.6% - Test accuracy: 80.0% - Validation AUC 0.887 with CI [0.824–0.951] Test AUC 0.819 with CI [0.685–0.954]
Logistic regression	- Training accuracy: 80.6% - Validation accuracy: 83.2% - Test accuracy: 63.3% - Validation AUC 0.845 with CI [0.772–0.918] Test AUC 0.667 with CI [0.505–0.829]
Random forest	- 500 estimators - Out-of-bag score estimate: 77.8% - Mean validation accuracy score: 84.3% - Mean test accuracy score: 76.7% - Validation AUC 0.855 with CI [0.783–0.926] - Test AUC 0.792 with CI [0.653–0.930]

*ML* machine learning; *DNN* deep neural network; *SGD* stochastic gradient descent; *AUC* area under the curve; *CI* confidence interval.

## Discussion

To the best of our knowledge, this study is the first to use machine learning to predict the need for AFO in stroke patients. AFO is one of the most frequently prescribed braces for the rehabilitation of stroke patients with gait disturbance^21^. The tibialis anterior is one of the muscles that contributes most to ankle flexion, and it is one of the muscles that commonly experiences motor impairment in patients with gait disturbance^22^. In a normal gait, the tibialis anterior is activated during the loading and swing phases^23^. During the swing phase, the activity of the tibialis anterior lifts the foot and toe to obtain foot clearance^23^. In general, AFO can improve foot clearance during the swing and stance phases^24^.

The most noticeable improvements occur in the first few weeks after the onset of stroke, then the rate of improvement slows and reaches a relatively stable state after 3 months^25–27^. Within 3 months after stroke onset, 70% of recovery in motor function is known to occur^28^. After 6 months, recovery usually reaches its limit and enters a chronic phase^29^. Therefore, in this study, we used the MRC score for ankle dorsiflexion at 6 months after stroke onset as an indicator of the need for AFO in stroke patients.

Machine learning models have been used to predict motor or cognition recovery in stroke patients^12–16^. For example, Lin et al. have investigated whether machine learning models can predict the recovery of activities of daily living in acute stroke patients^14^. They recruited 313 subjects and predicted the Barthel Index score at discharge using machine learning methods such as logistic regression, support vector machine, and random forest. The average of the AUC for the classification models (logistic regression, support vector machine, and random forest) were 0.755, 0.777 and 0.769 respectively. Other studies evaluated whether machine learning models could predict motor or cognition improvement in the acute and subacute stages of stroke^13, 15, 16^. Heo et al. have predicted the modified Rankin Scale score using deep neural network, logistic regression, and random forest with 2604 acute ischemic stroke subjects, and report AUCs of 0.888, 0.849, and 0.857, respectively^13^. Sale et al. have studied the predictability of improving motor and cognitive function after rehabilitation treatment from the early stages of stroke. They used data of 55 patients collected at the time of admission to the Department of Rehabilitation Medicine and at discharge, and predicted the Barthel Index and functional independence measure score with a linear support vector machine regression model. All output results and the actual measured results show a good correlation of 0.75–0.81^15^. Wang et al. have constructed a prognostic model of functional outcome using data from 333 patients with primary intracerebral hemorrhage. They utilized Auto-WEKA 2.0 that uses a sequential model-based algorithm configuration to determine the class with the best performance on the given data. Functional scores at 1 and 6 months after onset evaluated with the modified Rankin Scale were used as the outcome data. They show that the AUC predicting a 1-month outcome is 0.899, and the AUC predicting a 6-month outcome is 0.917^16^. The results of these studies are promising, with moderate to high accuracy. Similar to these previous studies, current study has demonstrated that machine learning models could accurately predict the need for AFO in acute stroke patients. Bearing in mind that AUCs of 0.7–0.8, 0.8–0.9, and > 0.9 are generally considered acceptable, excellent, and outstanding, respectively^30^, the ability of the machine learning models used in this study to predict the need for AFO is excellent, with the deep neural network model performing better than the other models (random forest and logistic regression models).

The deep neural network model may be more appropriate for predicting clinical outcomes^31^. Multiple layers of complex networks may be efficient for representing the complex characteristics of the clinical outcomes in a stroke patient^13^. However, the theoretical background underlying the improved performance reported for the deep neural network is unknown^32^. However, given that machine learning models can learn independently with additional data, the previously mentioned results could be improved^33^.

### Limitations

There are some limitations to this study. First, this was a single-center study, and should be verified with data from other sources. Second, variables used as inputs in machine learning algorithms are usually variables that can be acquired or evaluated in most cases. However, the prediction may be slightly affected by variables and may be adjusted to account for availability when considering data from different centers.

## Conclusion

This study demonstrated that machine learning algorithms, particularly the deep neural network, can improve the prediction of the need for AFO in acute stroke patients.
